# Lactic Acid Bacteria: An Inexhaustible Source of Scientific Knowledge and Food Innovation

**DOI:** 10.3390/foods14050858

**Published:** 2025-03-03

**Authors:** Luis M. Medina, Fernando Pérez-Rodríguez

**Affiliations:** Food Science and Technology Department, ENZOEM, Competitive Research Unit on Zoonoses and Emerging Diseases of the University of Cordoba, International Agrifood Campus of Excellence (ceiA3), Charles Darwin Annex Building, Campus de Rabanales, E-14071 Cordoba, Spain; b42perof@uco.es

For thousands of years, lactic acid bacteria (LAB) have been involved in food processes that have given rise to a wide range of traditional foods, mainly fermented foods. Their empirical formulation in food production has led to important innovations in the sector, particularly from the 1980s and 1990s onwards.

In the 1990s, conferences and congresses dedicated specifically to LAB, their role, and potential applications in the food industry became frequent, often with the dairy industry at the forefront.

Today, LAB are still the subject of continuous research and innovative applications, which, in parallel with the development of omics, have enabled the investigation of viable, but nonculturable bacteria (VBNC), and low prevalent microbial groups and their consequences in the configuration of microbial ecosystems. These advances have bridged the gap between taxonomies, taking into account the genomic diversity of the genus *Lactobacillus*, enriching and updating the perspectives from which they are studied.

The leading role of LAB in the world of probiotics, their ability to produce bacteriocins and other bioactive substances, combined with a human microbiome-based knowledge, bring about a wide range of applications of prime interest for these bacteria, which continues to be one of the most fertile fields of knowledge in food science.

This Special Issue looks at some of these future perspectives.

**Overview of contents of the Special Issue**

This Special Issue comprises ten contributions, seven of them research articles and three reviews. These works have been carried out in diverse universities and research centers of six different countries.

The review articles provide updates on very relevant aspects related to LAB, and in particular, delineate the future directions for their respective topics.

Without a doubt, LAB have played a role in fermentation processes for millennia, and Huang et al. [1], highlight the limitations and challenges of applying microbial fermentation in lactic acid production. Not without their own challenges, the authors report useful solutions for the industrial production of lactic acid.

Furthermore, LAB can also present biotechnological features for the production of other compounds, such as mannitol, a sugar alcohol, which is of interest due to its potential to reduce the use of sugars, given its low calories content. This is the case of *Leuconostoc citreum*, a promising microorganism for this purpose, which has been revised by Rosca et al. [2], especially for its use in sourdough-based baked products, extending into other benefits from its application.

A Special Issue devoted to LAB and future trends regarding them must always include new perspectives on probiotics. In this Special Issue, a review on the use of probiotics is presented, underscoring the need to demonstrate their safety when they are intended for use by pregnant women or during lactation. This topic has been revised by Fernández et al. [3].

Regarding the possibilities of applying LAB to control pathogens in foods, this Special Issue demonstrates the effects of selected autochthonous LAB (two *Lacticaseibacillus casei* strains) against *Listeria monocytogenes*. This work was carried out by Martín-Miguelez et al. [4] in two elaborations of soft-ripened cheese. The use of these strains, alone or combined, led to reductions of 2 to 4 log CFU/g in *L. monocytogenes* over 60 days of ripening, which could be a suitable strategy for the biocontrol of pathogen contamination during cheese processing. Furthermore, in another article [5], this research team reported that these selected LAB strains improved the sensory profile of the soft-ripened cheeses, decreasing the values of texture parameters such as hardness, gumminess, and chewiness for a softer texture, and increasing the umami taste and floral and lactic odor attributes. A sensory analysis revealed that consumers perceived differences between inoculated and non-inoculated cheeses, although the overall acceptability was not affected.

Sometimes, innovation can arise from the valorization of resources that have been forgotten or unused for years. Alba et al. [6] isolated and characterized a strain of *Ligilactobacillus salivarius* (*L. salivarius* SP36) from a cheese from a Pyrenean village sealed in 1936 and produced via household production. Overall, the phenotypic and genetic features of this strain support its high potential as an adjunct culture in cheesemaking. Other work in this Special Issue addresses the aforementioned aspects. Arias et al. [7] report that *L. salivarius* SP36 increased the formation of 25 volatile compounds, receiving higher scores for aroma intensity and quality than control cheeses.

When considering cheeses as food matrices, among the key attributes that make artisanal cheeses of particular value are their diverse aromatic profiles that impart distinctive characteristics to each product. In their article, Ruiz et al. [8] report the relationship between the activity of the autochthonous lactic bacteria (*Lacticaseibacillus paracasei*, *Lactiplantibacillus plantarum*, *Leuconostoc mesenteroides*, and *Lactococcus lactis* subsp. *hordniae*) involved in raw sheep milk cheeses, with the volatile compounds determined by GC-IMS, a technique with which these authors have been working for the last decade. The occurrence of *Lactococcus lactis* subsp. *hordniae* from cheese was reported for the first time.

Some of the most novel contributions to bacteriocin studies are represented in this Special Issue by the work of Feito et al. [9], a group led by Prof. Hernández-Cruza, from the Complutense University in Madrid (Spain). The work describes the application of *Lactococcus lactis* strains producing Garvicin A and/or Garvicin Q, either alone or together with Nisin A or Nisin Z, and high antimicrobial activity against *Lactococcus garvieae*, a main ichthyopathogen in rainbow trout (*Oncorhynchus mykiss*, Walbaum) farming.

As for the use of novel LAB found in natural sources such as fermented foods, Nazareth et al. [10] describe the isolation, identification, characterization, and quantification of LAB with antifungal activity, in this case in dry-cured sausages, for application in the field of meat products. The authors found promising biological candidates corresponding to *Lactiplantibacillus plantarum* and *Pediococcus pentosaceus*. *P. pentosaceus* C15 was distinguished from other bacteria thanks to its production of antifungal compounds such as nonanoic acid and phenyl ethyl alcohol, as well as its higher production of lactic and acetic acid.

We therefore present ten relevant examples of the inexhaustible source of scientific knowledge and innovative applications of lactic acid bacteria in product development, health, and food safety.
